# The Controversy About the Effects of Different Doses of Corticosteroid Treatment on Clinical Outcomes for Acute Respiratory Distress Syndrome Patients: An Observational Study

**DOI:** 10.3389/fphar.2021.722537

**Published:** 2021-07-29

**Authors:** Jia-Wei Yang, Ping Jiang, Wen-Wen Wang, Zong-Mei Wen, Bei Mao, Hai-Wen Lu, Li Zhang, Yuan-Lin Song, Jin-Fu Xu

**Affiliations:** ^1^ Department of Respiratory and Critical Care Medicine, Shanghai Pulmonary Hospital, School of Medicine, Institute of Respiratory Medicine, Tongji University, Shanghai, China; ^2^ Department of Anesthesiology, Shanghai Pulmonary Hospital, School of Medicine, Tongji University, Shanghai, China; ^3^ Department of Pulmonary Medicine, Zhongshan Hospital, Fudan University, Shanghai, China

**Keywords:** acute respiratory distress syndrome, corticosteroid, high-dose, interleukin-18, mortality

## Abstract

**Background:** Corticosteroid usage in acute respiratory distress syndrome (ARDS) remains controversial. We aim to explore the correlation between the different doses of corticosteroid administration and the prognosis of ARDS.

**Methods:** All patients were diagnosed with ARDS on initial hospital admission and received systemic corticosteroid treatment for ARDS. The main outcomes were the effects of corticosteroid treatment on clinical parameters and the mortality of ARDS patients. Secondary outcomes were factors associated with the mortality of ARDS patients.

**Results:** 105 ARDS patients were included in this study. Corticosteroid treatment markedly decreased serum interleukin-18 (IL-18) level (424.0 ± 32.19 vs. 290.2 ± 17.14; *p* = 0.0003) and improved arterial partial pressure of oxygen/fraction of inspired oxygen (PaO_2_/FiO_2_) (174.10 ± 65.28 vs. 255.42 ± 92.49; *p* < 0.0001). The acute physiology and chronic health evaluation (APACHE II) score (16.15 ± 4.41 vs. 14.88 ± 4.57, *p* = 0.042) decreased significantly on the seventh day after systemic corticosteroid treatment. Interestingly, the serum IL-18 decreased significantly (304.52 ± 286.00 vs. 85.85 ± 97.22, *p* < 0.0001), whereas the improvement of PaO_2_/FiO_2_ (24.78 ± 35.03 vs. 97.17 ± 44.82, *p* < 0.001) was inconspicuous after systemic corticosteroid treatment for non-survival patients, compared with survival patients. Furthermore, the receiver operating characteristic (ROC) model revealed, when equivalent methylprednisolone usage was 146.5 mg/d, it had the best sensitivity and specificity to predict the death of ARDS. Survival analysis by Kaplan–Meier curves presented the higher 45-day mortality in high-dose corticosteroid treatment group (logrank test *p* < 0.0001). Multivariate Cox regression analyses demonstrated that serum IL-18 level, APACHE II score, D-dimer, and high-dose corticosteroid treatment were associated with the death of ARDS.

**Conclusion:** Appropriate dose of corticosteroids may be beneficial for ARDS patients through improving the oxygenation and moderately inhibiting inflammatory response. The benefits and risks should be carefully weighed when using high-dose corticosteroid for ARDS.

**Trial registration:** This work was registered in ClinicalTrials.gov. Name of the registry: Corticosteroid Treatment for Acute Respiratory Distress Syndrome. Trial registration number: NCT02819453. URL of trial registry record: https://register.clinicaltrials.gov.

## Introduction

Acute respiratory distress syndrome (ARDS) is caused by an acute inflammatory injury to the lung, associated with increased pulmonary vascular permeability. Previous studies have characterized ARDS as a process of ongoing inflammation, parenchymal-cell proliferation, and disordered collagen deposition (Ware and Matthay, 2000; Ranieri et al., 2012; Matthay et al., 2019). Nevertheless, ARDS remains difficult to define, it is relatively underreported by clinicians, and its therapeutic options remain controversial (Ferguson et al., 2005; Hernu et al., 2013; Neto et al., 2016; Fan et al., 2018). Previous studies have reported that the hospital mortality of ARDS ranged from 34.9 to 46.1% (Rubenfeld et al., 2005; Villar et al., 2011; Bellani et al., 2016). However, declining mortality was observed over the past decades due to many efforts in the management of ARDS, such as advances in the application of mechanical ventilation and the use of adjunctive interventions in routine clinical practice (Zhang et al., 2019).

ARDS is a complicated response to pulmonary and systemic inflammatory responses involving neutrophil activation, alveolar epithelial, and vascular endothelial injury, leading to non-cardiogenic pulmonary edema and atelectasis (Choi et al., 2014). Systemic corticosteroids have been considered as a potential therapy for ARDS due to their anti-inflammatory and immunomodulatory effects. The improvement in oxygenation and reduction of the duration of mechanical ventilation in ARDS patients followed by corticosteroids administration have been reported in previous randomized controlled clinical trials (RCTs), but there was no conclusive evidence of lower mortality in these patients (Meduri et al., 1998; Steinberg et al., 2006; Meduri et al., 2007). Published meta-analyses on the use of corticosteroid treatment for ARDS also demonstrated inconsistent conclusions (Tang et al., 2009; Ruan et al., 2014; Meduri et al., 2016; Yang et al., 2017). In addition, the role of corticosteroids in ARDS induced by COVID-19 is controversial (Li et al., 2020; Liu et al., 2020; Wu et al., 2020; Horby et al., 2021; Tomazini et al., 2020). Recently, a published RCT has revealed that early administration of dexamethasone could reduce the duration of mechanical ventilation and overall mortality of moderate-to-severe ARDS patients (Villar et al., 2020). However, the dose, duration, and timing of corticosteroid administration for ARDS patients remain controversial, which has prompted us to do further research.

IL-18 was crucial for ARDS pathogenesis in previous studies (Dolinay et al., 2012; Makabe et al., 2012; Rogers et al., 2019). We aimed to determine the effects of varying doses of corticosteroids on IL-18 and explore the correlation between the different doses of corticosteroid administration and the prognosis of ARDS patients. This relationship is expected to have a certain guiding effect on clinical practice regarding determining the corticosteroid doses when treating patients with ARDS.

## Methods

### Patient Population

We enrolled adult patients diagnosed with ARDS admitted from July 2016 to December 2017 at Shanghai Pulmonary Hospital (Shanghai, China) by two clinicians. Inclusion criteria were as follows: 1) participants or their first-degree kin able to provide written informed consent; 2) aged 18–85 years; 3) confirmed diagnosis of ARDS by Berlin criteria (Ranieri et al., 2012). Exclusion criteria were as follows: 1) active tuberculosis and disseminated fungal infection; 2) chronic corticosteroid application; 3) patients with organ dysfunction, such as severe liver dysfunction, adrenal insufficiency, and severe cardiopulmonary dysfunction; 4) hypogammaglobulinemia or other autoimmune diseases; 5) acquired immunodeficiency syndrome; 6) refusing to use corticosteroids; 7) pregnant or nursing.

### Study Design

This is an observational study. All patients came from the departments of emergency, respiratory, and critical care medicine. The investigators who were responsible for collecting the clinical records and the follow-up, statisticians, and the physicians who assigned treatment were not the same person. Two clinicians assessed and decided the clinical diagnosis and therapy for these patients. If the diagnosis and treatment strategy of the two clinicians differed, a decision was reached by consensus. When clinicians initially diagnosed ARDS on the first day of hospital admission and decided to use systemic corticosteroid treatment, blood samples of these patients were collected to detect the concentration of IL-18 before treatment and taken again on the fourth day after systemic corticosteroid treatment. We recorded the 45-day mortality after hospital admission by telephone or face-to-face interviews. This study was approved by the Clinical Research Ethics Board of Shanghai Pulmonary Hospital, Tongji University (K14-152). Informed consent was obtained from participants or their first-degree kin when they were recruited into the study.

### Intensive Care Unit Admission and Corticosteroid Treatment

Patients with sepsis, moderate-to-severe ARDS, multiple organ dysfunction syndrome (MODS), and requiring mechanical ventilation were admitted to the intensive care unit (ICU) unless patients or their first-degree kin refused. Corticosteroid allocation differed among patients whose treatment policies were decided according to their individual situations. Patients received an initial higher dose of corticosteroid with a tapering regimen according to their condition assessed by two clinicians. Certainly, in some patients, the dose of corticosteroid may be increased during the course of treatment and the course of treatment could be prolonged based on their own conditions.

### Human Sample Collection and Processing

A 5 ml of whole blood samples from ARDS patients was collected in pro-coagulation tube at baseline and after receiving intravenous corticosteroids on the fourth day. The blood samples were instantly centrifuged (10 min, 3000 rpm at 4°C) and then separated serum samples were kept frozen at −80°C until further analysis. IL-18 levels in serum were detected using a human IL-18 enzyme-linked immunosorbent assay (ELISA) kit (eBioscience BMS267/2).

### Variables

The following variables were collected: 1) demographics data, age and sex; 2) underlying diseases; 3) severity of illness, APACHE II score and SOFA score were assessed at initial diagnosis of ARDS, on the third day, and on the seventh day after treatment and the proportion of the patients who had mild, moderate or severe ARDS in each treatment arm were also assessed; 4) serological indicators, C-reactive protein (CRP), brain natriuretic peptide (BNP), D-dimer, lactic dehydrogenase (LDH), procalcitonin (PCT), neutrophils/lymphocyte (N/L), serum IL-18 and PaO_2_/FiO_2_ before and after corticosteroid treatment; 5) treatments and outcomes.

### Statistics Analysis

Continuous variables were described by mean ± SD (standard deviation) or median with interquartile range (IQR, 25–75%), as appropriate. Categorical variables were described by absolute numbers (percentages). Student’s *t*-test and Mann–Whitney *U*-test were used to analyze normally and non-normally distributed continuous data, respectively. Categorical variables were analyzed by the chi-square test. All tests of significance were two-tailed and a *p* value < 0.05 was considered statistically significant. We used the receiver operating characteristic (ROC) curve to evaluate the accuracy of corticosteroid application predicted mortality. When the area under curve (AUC) is more than 0.7, it indicates that corticosteroid application has better prediction accuracy for mortality. The sensitivity and specificity of various doses of corticosteroid application to predict mortality were analyzed to define the cut-off value of dosage of corticosteroid used. Independent factors affecting patient survival were determined using univariable analysis and multivariate Cox regression models. The adjusted hazard ratios (HR) of the variables incorporated into the model and their 95% confidence intervals were calculated when HR > 1 indicated a higher probability of death. The survival curve of 45-day mortality for patients between high- and low-dose corticosteroid treatment groups was analyzed by the Kaplan–Meier method and compared by logrank test.

The statistical package SPSS (version 19.0; SPSS, Chicago, IL, United States) was used for statistical analysis, and GraphPad Prism (version 8; GraphPad Software, San Diego, CA, United States) used for drawing graphs.

## Results

### Clinical Characteristics

A total of 105 ARDS patients caused by severe pulmonary infection, who all received systemic corticosteroid treatment, were included in this study. 85 patients (80.95%) were male, The APACHE II score was 15.52 ± 5.08, and the SOFA score was 4.90 ± 1.94 at baseline. 67 patients (63.81%) were admitted to the ICU and 47 patients (44.76%) received mechanical ventilation. Overall hospital mortality was 23/105 (21.9%), and ICU mortality was 23/67 (34.33%). Larger doses of corticosteroid [175 (148–240) mg/d vs. 88.5 (53–123) mg/d, *p* = 0.001] tend to be used in the non-survival group. More patients received mechanical ventilation (22/23 vs. 25/82, *p* < 0.0001) and the length of mechanical ventilation was longer in the non-survival group than that in the survival group (10.18 ± 9.50 days vs. 5.68 ± 4.09 days, *p* = 0.049). The common complications were superinfection and hyperglycemia after corticosteroid treatment. Compared with the survival group, there was more superinfection (9/23 vs. 11/82, *p* = 0.013) in the non-survival group. There was no difference in hyperglycemia between the two groups (16/23 vs. 39/82, *p* = 0.097) (Table 1).

**TABLE 1:** Baseline characteristics and outcomes after corticosteroid treatment for patients with acute respiratory distress syndrome.

	Non-survival (*n* = 23)	Survival (*n* = 82)	Total (*n* = 105)	*p* value
Age, yr	63.09 ± 12.68	58.87 ± 12.58	59.79 ± 12.67	0.159
Male gender	19 (82.61)	66 (80.49)	85 (80.95)	1.000
Underlying diseases	—	—	—	—
Cardiovascular diseases	14 (60.87)	34 (41.46)	48 (45.71)	0.099
Diabetes	8 (34.78)	18 (21.95)	26 (24.76)	0.208
Respiratory diseases	8 (34.78)	25 (30.49)	33 (31.43)	0.695
Others	11 (47.83)	26 (31.71)	37 (35.24)	0.216
Severity of illness	—	—	—	—
Apache II score	19.09 ± 5.28	14.52 ± 4.58	15.52 ± 5.08	0.001
SOFA score	6.26 ± 2.53	4.52 ± 1.56	4.90 ± 1.94	<0.001
ICU admission	23 (100)	44 (53.66)	67 (63.81)	<0.001
Mild	0 (0)	38 (46.34)	38 (36.19)	<0.001
Moderate	10 (43.48)	40 (48.78)	50 (47.62)	0.814
Severe	13 (56.52)	4 (4.88)	17 (16.19)	<0.001
Laboratory index	—	—	—	—
BNP (ng/ml)	1,073.74 ± 1921.54	381.45 ± 490.83	533.10 ± 1,025.41	0.100
CRP (mg/L)	92.92 ± 53.88	55.85 ± 52.88	63.97 ± 55.04	0.004
PCT (ng/ml)	1.12 ± 1.43	0.28 ± 0.37	0.46 ± 0.81	0.010
LDH (U/L)	767.96 ± 531.20	517.48 ± 243.47	572.34 ± 341.61	0.038
D-dimer (ng/ml)	4,017.78 ± 4,662.70	1,412.60 ± 1709.62	1983.26 ± 2,836.83	0.015
PaO_2_/FiO_2_ (mmHg)	104.83 ± 38.64	193.54 ± 57.62	174.14 ± 65.28	<0.001
IL-18 (pg/ml)	802.26 ± 476.44	317.84 ± 161.30	423.95 ± 329.86	<0.001
Neutrophils/lymphocyte	12.01 ± 5.59	9.33 ± 5.23	9.92 ± 5.40	0.035
Systemic steroid treatment	—	—	—	—
Equivalent methylprednisolone (mg/d) median (IQR)	175 (148–240)	88.5 (53–123)	120 (100–160)	0.001
Length of steroid (d) median (IQR)	11 (4–17)	12 (8–15)	12 (7–16)	0.574
Mechanical ventilation	22 (95.65)	25 (30.49)	47 (44.76)	<0.001
Length of mechanical ventilation(d)	10.18 ± 9.50	5.68 ± 4.09	7.79 ± 7.42	0.049
Length of hospital stay(d)	13.48 ± 9.71	14.73 ± 6.80	14.46 ± 7.50	0.566
Complications	—	—	—	—
Superinfection	9 (39.13)	11 (13.41)	20 (19.05)	0.013
Hyperglycemia	16 (69.57)	39 (47.56)	55 (52.38)	0.097

APACHE II: acute physiology and chronic health evaluation; BNP: brain natriuretic peptide; CRP: C-reactive protein; PaO_2_/FiO_2_: arterial partial pressure of oxygen/fraction of inspired oxygen; IL-18: interleukin-18; LDH: lactic dehydrogenase; PCT: procalcitonin.

The continuous variables are presented as the mean ± standard deviation (SD) or medians (interquartile ranges), and the categorical variables are presented as the numbers (percentages).

### Outcomes of Patients

**FIGURE 1:**
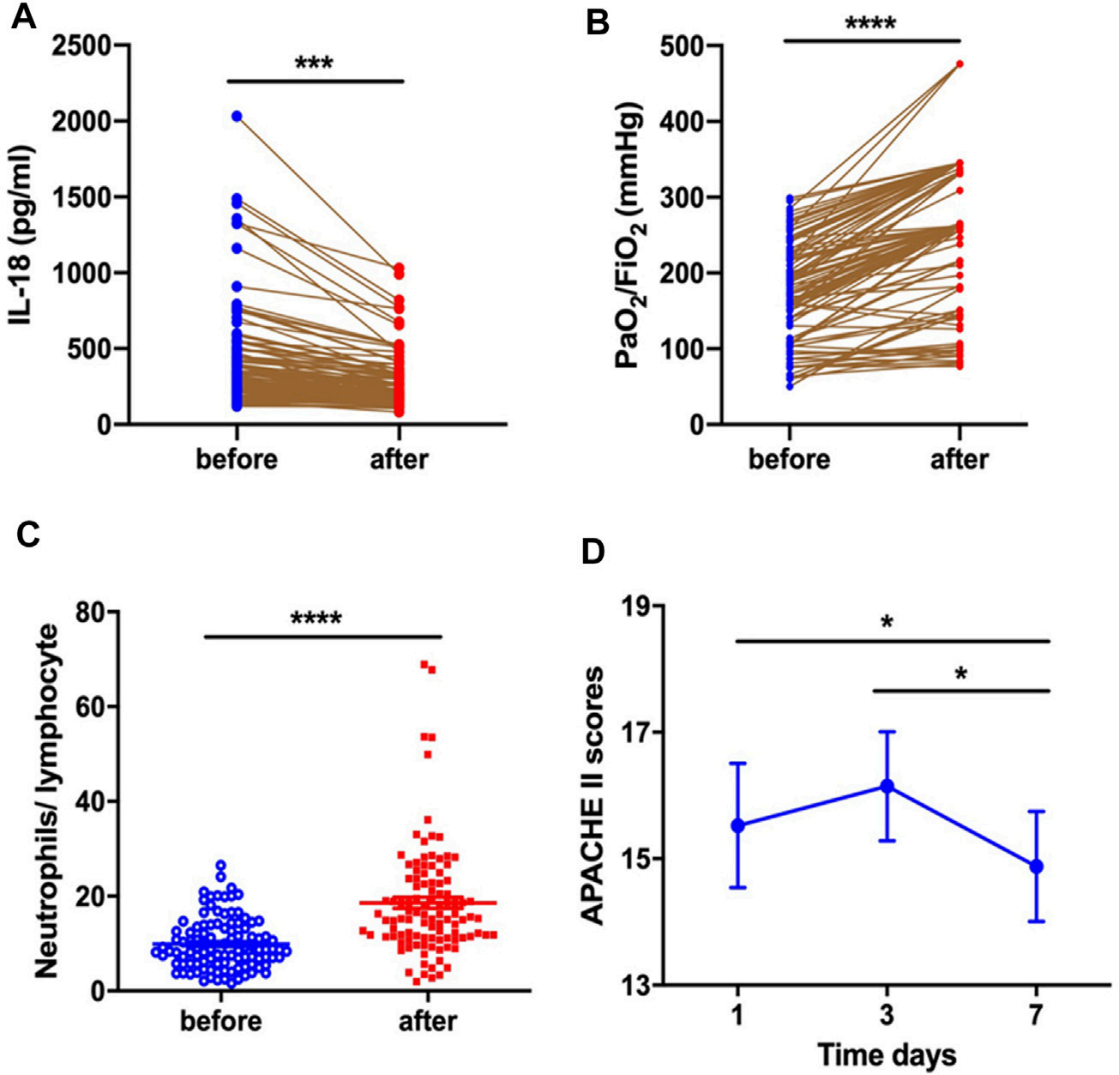
Corticosteroid treatment decreased serum IL-18 level **(A)** and improved PaO_2_/FiO_2_ **(B)**, increased the neutrophil-to-lymphocyte ratio **(C)**, and decreased APACHE II score **(D)** at the seventh day.

Serum IL-18 levels markedly decreased (424.0 ± 32.19 vs. 290.2 ± 17.14, *p* = 0.0003) (Figure 1A) and PaO_2_/FiO_2_ improved (174.10 ± 65.28 vs. 255.42 ± 92.49, *p* < 0.0001) (Figure 1B) for ARDS patients after systemic corticosteroid treatment. A significant increase for N/L was observed after corticosteroid treatment (9.92 ± 5.40 vs. 18.58 ± 11.87, *p* < 0.0001) (Figure 1C). Whether compared with baseline (15.52 ± 5.08 vs. 14.88 ± 4.57, *p* = 0.049) or the third day after treatment (16.15 ± 4.41 vs. 14.88 ± 4.57, *p* = 0.042), systemic corticosteroid treatment decreased APACHE II score at the seventh day (Figure 1D).

### Outcomes in the Survival and Non-Survival Group

**FIGURE 2:**
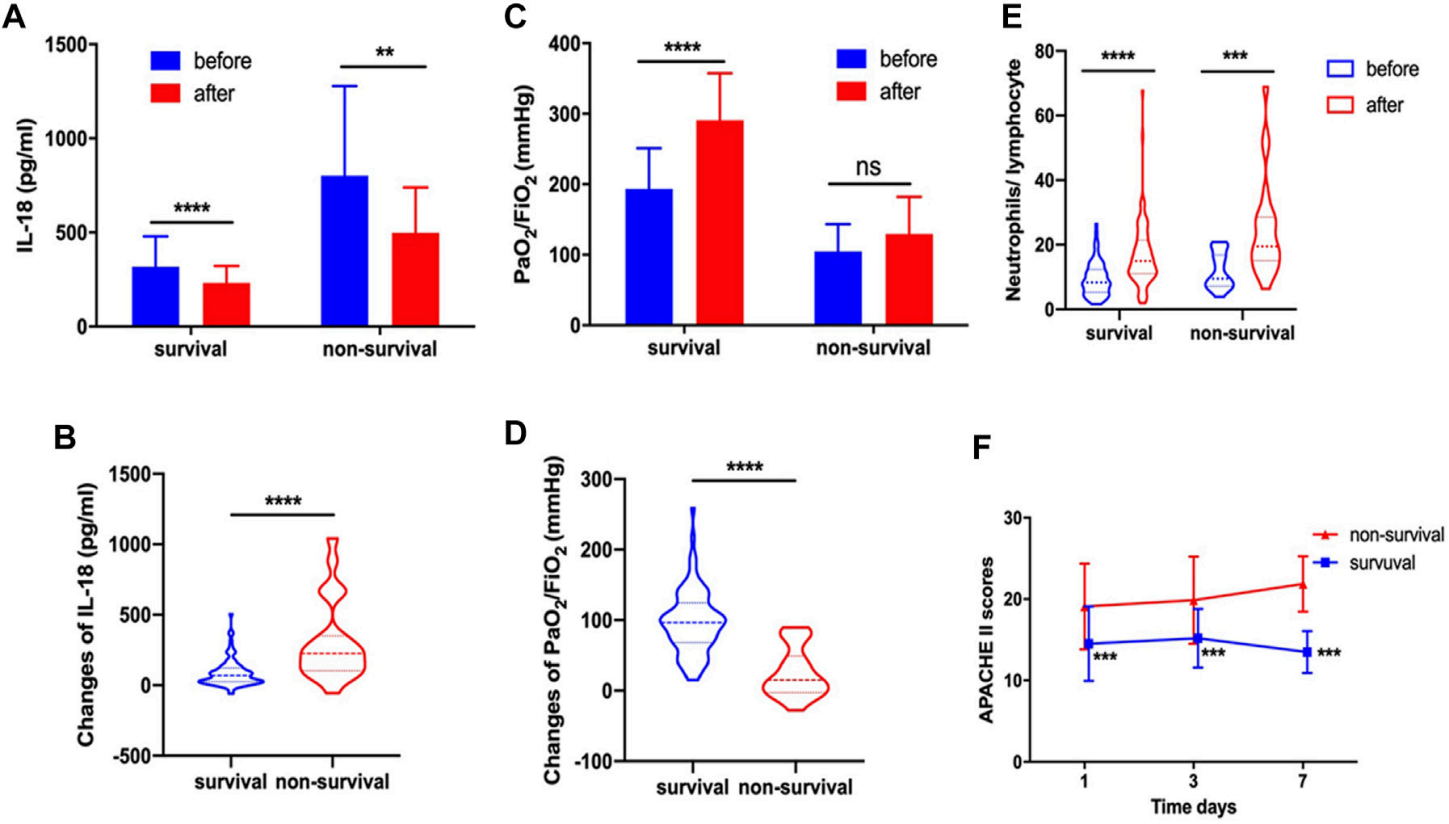
In survival and non-survival groups, corticosteroid treatment decreased serum IL-18 **(A)**. Changes of IL-18 after corticosteroid treatment **(B)**. Corticosteroid treatment improved PaO_2_/FiO_2_ **(C)**. Changes of PaO_2_/FiO_2_ after corticosteroid treatment **(D)**. Corticosteroid treatment increased the neutrophil-to-lymphocyte ratio **(E)**. APACHE II score at day 1, day 3, and day 7 **(F)**.

Serum IL-18 decreased significantly after systemic corticosteroid treatment in both survival and non-survival groups (Figure 2A). Further, the changes of IL-18 (304.52 ± 286.00 vs. 85.85 ± 97.22, *p* < 0.0001) were more pronounced in the non-survival group after corticosteroid therapy compared with the survival group (Figure 2B). Systemic corticosteroid treatment obviously improved the PaO_2_/FiO_2_ of patients in the survival group but not in the non-survival group (Figure 2C). Apparently, the improvement of PaO_2_/FiO_2_ was more significant in the survival group (97.17 ± 44.82 vs. 24.78 ± 35.03, *p* < 0.0001) (Figure 2D). We found that N/L increased significantly after corticosteroid treatment in both the survival group or the non-survival group (Figure 2E). Whether at baseline or during corticosteroid treatment, the APACHE II score of patients who died was always higher than that of those who survived (*p* < 0.001) (Figure 2F).

### Corticosteroid Treatment and Mortality

**FIGURE 3:**
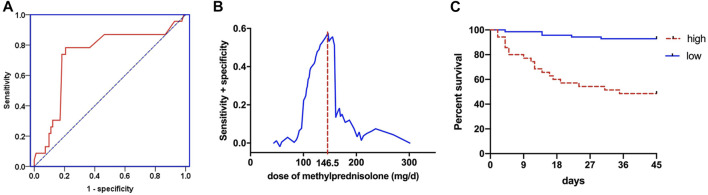
The regression model between corticosteroid treatment and mortality for ROC curve **(A)**. Sensitivity and specificity analysis of mortality in different doses of corticosteroid treatment **(B)**. Kaplan–Meier method to analyze the 45-day mortality **(C)** in the high-dose and low-dose corticosteroid treatment groups.

The regression model of corticosteroid treatment and mortality had an area under the ROC of 0.735, indicating the better propensity of death (Figure 3A). When equivalent to methylprednisolone usage of 146.5 mg/d, it has the highest sensitivity and specificity of predicting the death of ARDS patients (Figure 3B). Patients were assigned into high-dose and low-dose groups based on this point. The Kaplan–Meier curves revealed that the ARDS patients had the higher 45-day mortality in the high-dose group than that in the low-dose group (logrank test *p* < 0.0001) (Figure 3C).

### Outcomes in the High-Dose and Low-Dose Corticosteroid Treatment Groups

**FIGURE 4:**
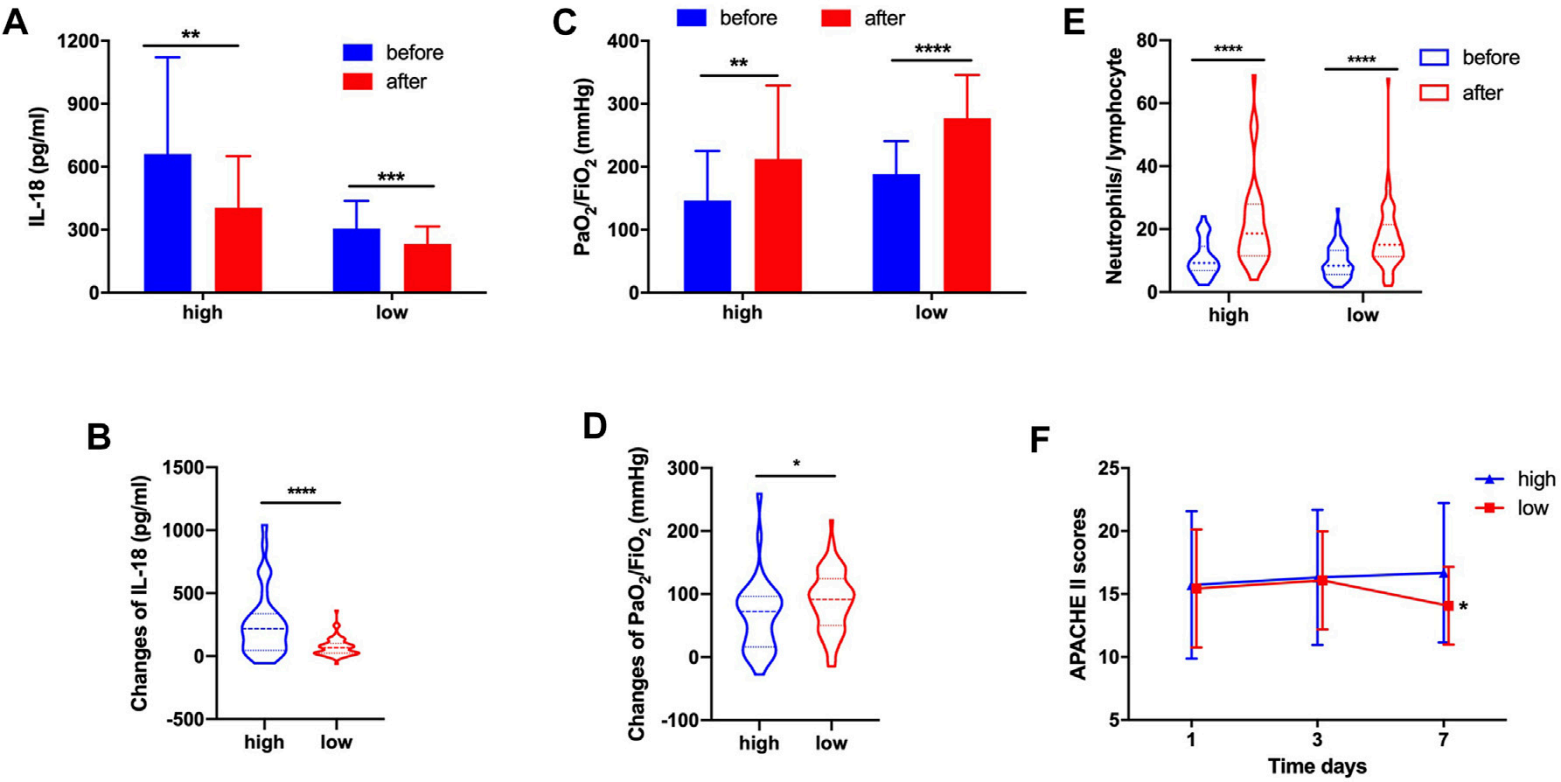
In high-dose and low-dose groups, corticosteroid treatment decreased serum IL-18 **(A)**. Changes of IL-18 after corticosteroid treatment **(B)**. Corticosteroid treatment improved PaO_2_/FiO_2_ **(C)**. Changes of PaO_2_/FiO_2_ after corticosteroid treatment **(D)**. Corticosteroid treatment increased the neutrophil-to-lymphocyte ratio **(E)**. APACHE II score at day 1, day 3, and day 7 **(F)**.

Similarly, we found that whether in the high-dose group or low-dose group, the serum IL-18 significantly decreased after systemic corticosteroid treatment (Figure 4A). Obviously, the changes of IL-18 were also more significant in the high-dose corticosteroid group (255.43 ± 260.27 vs. 72.91 ± 70.51, *p* < 0.0001) (Figure 4B). High-dose and low-dose systemic corticosteroid treatment can obviously improve the oxygenation of ARDS patients (Figure 4C). Interestingly, the changes of oxygenation were more pronounced in the low-dose corticosteroid treatment group (65.86 ± 57.82 vs. 89.04 ± 47.80, *p* = 0.031) (Figure 4D). The N/L increased significantly after high-dose and low-dose corticosteroid treatment (Figure 4E). It revealed that the APACHE II score of the low-dose group was significantly lower than that in the high-dose group at the seventh day after systemic corticosteroid treatment (Figure 4F).

### Factors Associated With Mortality

Results of univariate analysis and multivariate Cox regression analyses are shown in Table 2. In this model, we found that after adjusting for confounding factors, high-dose corticosteroid treatment was an independent risk factor for death of ARDS patients (adjusted HR 5.939, 95% CI: 1.925–18.322, *p* = 0.002). Moreover, serum IL-18 at baseline (adjusted HR 1.002, 95% CI: 1.001–1.004, *p* = 0.000), APACHE II score (adjusted HR 1.172, 95% CI: 1.052–1.305, *p* = 0.004), and D-dimer (adjusted HR 1.016, 95% CI: 1.005–1.027; *p* = 0.005) were also significantly associated with the increased mortality (Table 2).

**TABLE 2:** Variables associated with death, as shown in univariable and multivariate Cox regression analysis.

Variables	HR (95% CI)	*p* value	Adjusted HR (95% CI)	*p* value
Sex	2.582 (1.131–5.893)	0.024	1.234 (0.477–3.195)	0.664
N/L	1.071 (1.002–1.145)	0.045	1.046 (0.966–1.133)	0.269
IL-18	1.004 (1.003–1.005)	0.000	1.002 (1.001–1.004)	0.000
D-dimer (per 100 ng/ml)	1.017 (1.008–1.026)	0.000	1.016 (1.005–1.027)	0.005
APACHE II score	1.223 (1.108–1.349)	0.000	1.172 (1.052–1.305)	0.004
Corticosteroid (high)	9.683 (3.584–26.159)	0.000	5.939 (1.925–18.322)	0.002

APACHE II: acute physiology and chronic health evaluation; CI: confidence interval; HR: hazard ratio; IL-18: interleukin-18; N/L: the ratio of neutrophils/lymphocyte.

HR (95% CI) represent univariable Cox regression analysis.

Adjusted HR (95% CI) represent multivariate Cox regression analysis.

## Discussion

Inflammation was known to exert an important role in the development of ARDS and had an impact on the prognosis of ARDS. Cox regression analysis in our study presented that serum IL-18 level at baseline was an independent risk factor for the mortality of ARDS patients. It was consistent with previous studies where IL-18 was crucial in the pathogenesis and prognosis of patients with ARDS (Dolinay et al., 2012; Makabe et al., 2012; Rogers et al., 2019). Intriguingly, we found that systemic corticosteroid treatment significantly decreased the level of serum IL-18. This finding may provide evidence for corticosteroid usage for ARDS patients. However, further research demonstrated that the decrease of serum IL-18 was more obvious in the non-survival group than in the survival group. Similarly, the decrease of IL-18 was more pronounced when treated with high-dose corticosteroid. The larger the dose of corticosteroid, the more obvious the inhibition of inflammatory response. When high-dose systemic corticosteroid administration produced an excessive suppression of the inflammatory response, a worse prognosis is predicted, leading to the dramatic reduction of the IL-18 level. Moreover, we found that systemic corticosteroid treatment improved oxygenation for ARDS patients. However, subgroup analysis showed that systemic corticosteroid treatment did not significantly improve the oxygenation of ARDS patients in the non-survival group. The improvements in oxygenation were more pronounced in the low-dose corticosteroid treatment group than that in the high-dose corticosteroid treatment group. These indicate that the improvement of oxygenation can predict the prognosis of ARDS patients and guide the dosage of corticosteroid usage.

Excessive release of cytokines and chemokines caused by neutrophil infiltration mediates the pathogenesis of ARDS, which may be associated with cytotoxicity, vascular stasis, and breaking the balance of pro-inflammatory and anti-inflammatory reactions. Studies have described the dual role of neutrophils in ARDS (Grudzinska and Sapey, 2018; Blázquez-Prieto et al., 2018). Moreover, when a large number of neutrophils were mobilized and activated and neutrophil-associated elastases were greatly released in bronchoalveolar lavage fluid (BALF), it was significantly associated with the severity and prognosis of ARDS (Summers et al., 2014; Bai et al., 2015). The timing of targeting neutrophils is suggested to be critical for improving the outcomes of ARDS. Moreover, several studies have reported the correlation between the N/L and the severity of the clinical course for ICU patients, suggesting that N/L should be considered a prognostic biomarker for ICU patients (Riché et al., 2015; Hwang et al., 2017; Wang et al., 2018). Song and colleagues have reported that N/L > 14 was associated with shorter overall survival of ARDS patients and it was an independent prognostic factor for overall survival (Wang et al., 2018). It prompted us to explore the treatment effect of corticosteroids on N/L of ARDS. Interestingly, our results have demonstrated that corticosteroid treatment significantly increased N/L in peripheral blood of ARDS patients. Further analysis has revealed that N/L increased significantly in the non-survival and the high-dose corticosteroid treatment groups, which was associated with the severity and poorer outcomes of patients. It may be a potential predictive prognostic biomarker for corticosteroid therapy in ARDS patients.

Our results have shown that the inflammatory parameters of CRP, PCT, LDH, and IL-18 were significantly higher in the non-survival group than those in the survival group, which presented more severe ARDS with the worse APACHE II score and SOFA score. They were responsible for the higher dose of corticosteroids used and more patients receiving mechanical ventilation; the longer length of mechanical ventilation duration was observed in the non-survival group. In addition, higher doses of corticosteroids were probably allocated to severe ARDS patients, and it was difficult to overcome this bias during the clinical practice completely. There is no strict guideline on the dosage and duration of corticosteroid therapy for ARDS patients worldwide. Similarly, corticosteroid treatment for COVID-19-induced ARDS remains controversial and without guidelines. Chen et al. have reported that administration of corticosteroids in severe COVID-19-related ARDS increased 28-day mortality and delayed SARS-CoV-2 coronavirus RNA clearance (Liu et al., 2020). However, RECOVERY research and Song et al. have reported that corticosteroid use for ARDS patients with COVID-19 resulted in lower mortality (Wu et al., 2020; Horby et al., 2021). Currently, the main controversies are concerning the timing, dosage, and duration of corticosteroid treatment for ARDS. Moreover, two RCTs on corticosteroid therapy for ARDS on the seventh day have reported opposite conclusions (Meduri et al., 1998; Steinberg et al., 2006; ). More studies were concerned with the early corticosteroid administration for ARDS patients (Meduri et al., 2007; Tang et al., 2009; Li et al., 2020; Liu et al., 2020; Villar et al., 2020). Recently, one RCT study has revealed that early corticosteroid treatment reduced the duration of mechanical ventilation and overall mortality for moderate-to-severe ARDS patients (Villar et al., 2020). However, some studies have reported the opposite conclusion. They revealed that early initiation (≤3 days from hospitalization or ICU admission) of corticosteroid therapy for COVID-19-induced ARDS was associated with higher 28-day mortality (Li et al., 2020; Liu et al., 2020). So far, there have been no RCTs on the dosage of corticosteroid therapy for ARDS. Takaki et al. have reported that initial high-dose methylprednisolone (1000 mg/d) with a tapering regimen had a negative effect on ARDS patients in a retrospective propensity-matched cohort study (Takaki et al., 2017). Our results have revealed that when the methylprednisolone equivalent dose was more than 146.5 mg/d, it was an independent risk factor of death and had the higher mortality of ARDS patients, which was consistent with the results of Chen et al.’s study. Their subgroup analysis demonstrated that high-dose (>200 mg equivalent hydrocortisone per day) corticosteroid therapy was associated with higher 28-day mortality rate of ARDS patients induced by COVID-19 (Liu et al., 2020). Of course, this phenomenon could be attributed to the fact that the used corticosteroid dosage was related to the severity of the disease; the more serious the disease is, the greater the dosage is.

The common adverse effects of corticosteroid therapy seem to be hyperglycemia and superinfection. However, the occurrence of corticosteroid-induced hyperglycemia was similar in the non-survival and survival groups. More superinfection was observed in the non-survival group, which reminded clinicians to be cautious about using large doses of corticosteroid therapy for severe ARDS patients.

## Limitations

This is a single-center study and limitations were unavoidable. In this study, corticosteroid treatment was evaluated in ARDS patients with severe pulmonary infection and intense inflammation. Therefore, we caution against extrapolating our findings to all types of ARDS patients. Secondly, there were few patients with ARDS who did not receive corticosteroid treatment during the study; thus, matching with the patients who received corticosteroid therapy was challenging. Therefore, a control group of no steroid treatment group was absent. Thirdly, there is no strict guideline on the dosage and duration of corticosteroid therapy for ARDS patients. Therapeutic strategies may differ among patients, whose treatment policies were decided according to their individual situations. Lastly, higher doses of corticosteroids were probably allocated to more severe patients, which led to some selection bias, although Cox regression analyses were performed to adjust for confounding factors. It was difficult to completely overcome during the clinical practice.

## Conclusion

In conclusion, the dosage of corticosteroids may be related to the prognosis of ARDS patients. The appropriate dose of corticosteroids improved the oxygenation of patients and alleviated IL-18-associated inflammation and the severity of ARDS, whereas high-dose corticosteroid was associated with higher mortality, because the influence of selection bias on the disease severity cannot be ruled out. Sufficiently powered RCT studies with rigorous control group to evaluate the effects of different doses of corticosteroid on ARDS are urgently warranted in the near future.

## Data Availability

The original contributions presented in the study are included in the article/Supplementary Material; further inquiries can be directed to the corresponding author.
